# Supplementary material for: Modeling glioblastoma heterogeneity as a dynamic network of cell states
Source: Mol Syst Biol. 2021 Sep 16;17(9):e10105. doi: 10.15252/msb.202010105 (PMC8444284; doi:10.15252/msb.202010105)
Supplement: Supplementary file 6 — Source Data for Figure 5 [file MSB-17-e10105-s004.zip › Figure5A_sourcedata/GSEA_3017/nosigdb_stateE.GseaPreranked.1622795031093/RICHARDS_NEFTEL_CELL_2019_G2_M.html]

Details for gene set RICHARDS\_NEFTEL\_CELL\_2019\_G2\_M[GSEA]

|  || Dataset | state63017 |
| Phenotype | NoPhenotypeAvailable |
| Upregulated in class | na\_neg |
| GeneSet | RICHARDS\_NEFTEL\_CELL\_2019\_G2\_M |
| Enrichment Score (ES) | -0.68731695 |
| Normalized Enrichment Score (NES) | -3.0663311 |
| Nominal p-value | 0.0 |
| FDR q-value | 0.0 |
| FWER p-Value | 0.0 |
Table: GSEA Results Summary

  

Fig 1: Enrichment plot: RICHARDS\_NEFTEL\_CELL\_2019\_G2\_M      
 Profile of the Running ES Score & Positions of GeneSet Members on the Rank Ordered List

  

| PROBE | GENE SYMBOL | GENE\_TITLE | RANK IN GENE LIST | RANK METRIC SCORE | RUNNING ES | CORE ENRICHMENT || 1 | TUBA1C |  |  | 282 | -0.253 | -0.3008 | No |
| 2 | OIP5 |  |  | 426 | -0.291 | -0.4468 | No |
| 3 | KIF20A |  |  | 523 | -0.321 | -0.5398 | No |
| 4 | GPSM2 |  |  | 550 | -0.334 | -0.5550 | No |
| 5 | TROAP |  |  | 562 | -0.341 | -0.5535 | No |
| 6 | HMGB3 |  |  | 577 | -0.348 | -0.5550 | No |
| 7 | RANGAP1 |  |  | 698 | -0.429 | -0.6701 | Yes |
| 8 | MZT1 |  |  | 711 | -0.437 | -0.6658 | Yes |
| 9 | TTK |  |  | 724 | -0.454 | -0.6609 | Yes |
| 10 | SPAG5 |  |  | 729 | -0.458 | -0.6470 | Yes |
| 11 | KNSTRN |  |  | 751 | -0.485 | -0.6507 | Yes |
| 12 | CDCA3 |  |  | 759 | -0.497 | -0.6385 | Yes |
| 13 | MXD3 |  |  | 763 | -0.502 | -0.6217 | Yes |
| 14 | NCAPD2 |  |  | 784 | -0.540 | -0.6221 | Yes |
| 15 | CDC20 |  |  | 793 | -0.562 | -0.6084 | Yes |
| 16 | RACGAP1 |  |  | 796 | -0.566 | -0.5879 | Yes |
| 17 | TUBB4B |  |  | 804 | -0.573 | -0.5727 | Yes |
| 18 | CDCA8 |  |  | 808 | -0.585 | -0.5525 | Yes |
| 19 | PLK1 |  |  | 809 | -0.586 | -0.5291 | Yes |
| 20 | FOXM1 |  |  | 814 | -0.593 | -0.5097 | Yes |
| 21 | AURKA |  |  | 824 | -0.623 | -0.4947 | Yes |
| 22 | CKAP2L |  |  | 826 | -0.625 | -0.4707 | Yes |
| 23 | BUB1 |  |  | 837 | -0.653 | -0.4556 | Yes |
| 24 | ANP32E |  |  | 841 | -0.676 | -0.4318 | Yes |
| 25 | ECT2 |  |  | 854 | -0.717 | -0.4163 | Yes |
| 26 | KIF2C |  |  | 855 | -0.721 | -0.3874 | Yes |
| 27 | KIF4A |  |  | 861 | -0.736 | -0.3634 | Yes |
| 28 | CCNB2 |  |  | 865 | -0.745 | -0.3369 | Yes |
| 29 | ARHGAP11A |  |  | 867 | -0.756 | -0.3077 | Yes |
| 30 | BUB1B |  |  | 871 | -0.766 | -0.2803 | Yes |
| 31 | CCNA2 |  |  | 872 | -0.770 | -0.2494 | Yes |
| 32 | TMPO |  |  | 875 | -0.778 | -0.2204 | Yes |
| 33 | CKAP2 |  |  | 878 | -0.781 | -0.1913 | Yes |
| 34 | KIFC1 |  |  | 883 | -0.807 | -0.1634 | Yes |
| 35 | H2AFZ |  |  | 893 | -0.849 | -0.1393 | Yes |
| 36 | KPNA2 |  |  | 895 | -0.858 | -0.1060 | Yes |
| 37 | SMC4 |  |  | 902 | -0.886 | -0.0771 | Yes |
| 38 | CCNB1 |  |  | 906 | -0.895 | -0.0445 | Yes |
| 39 | KIF11 |  |  | 913 | -0.957 | -0.0128 | Yes |
| 40 | CENPF |  |  | 935 | -1.200 | 0.0121 | Yes |
Table: GSEA details [plain text format]

  

Fig 2: RICHARDS\_NEFTEL\_CELL\_2019\_G2\_M: Random ES distribution      
 Gene set null distribution of ES for **RICHARDS\_NEFTEL\_CELL\_2019\_G2\_M**
